# Physical Intelligence in Small‐Scale Robots and Machines

**DOI:** 10.1002/adma.202510332

**Published:** 2025-10-17

**Authors:** Huyue Chen, Metin Sitti

**Affiliations:** ^1^ School of Medicine and College of Engineering Koç University Istanbul 34450 Türkiye; ^2^ Physical Intelligence Department Max Planck Institute for Intelligent Systems 70569 Stuttgart Germany

**Keywords:** implantable sensors, mechanical metamaterials, physical intelligence, robotics, smart materials

## Abstract

Intelligent living organisms—from unicellular entities to plants—rely on body physical intelligence (PI) to autonomously adapt and thrive in dynamic and complex environments, bypassing neural processing. The paradigm of PI has become a pivotal framework for small‐scale mobile robots and machines, where they have limited onboard powering, actuation, perception, computation, and control. However, the emerging PI capabilities remain rudimentary compared to biological counterparts in adaptability, multifunctionality, and evolvability. Here, the review systematically examines PI in small‐scale mobile robots and machines, highlight the importance of PI in extreme environments, elucidate hierarchical PI manifestations, identify current challenges and future opportunities for further promoting the evolution of PI. Notably, Current research emphasizes that the human body, featuring confined spaces, active and uncertain fluid and organ movements, immunological reactions, and heterogeneous physicochemical conditions, can be an ultimate testing ground for the next‐generation small‐scale robotic systems with more advanced PI. Looking forward, the rapid evolution of PI benefits from the convergence of multiple disciplines, such as robotics, mechanics, materials, chemistry, biology, and medicine, toward creating autonomous intelligent machines for real‐world applications.

## Graphical abstract

“Physical intelligence” (PI) empowers biological organisms and artificial machines, especially at the small scales, to perceive, adapt, and even reshape their complex, dynamic, and unstructured operation environments. This review summarizes recent milestones and future directions of PI in small‐scale robots and machines. As an example, programming actuation, computing, and sensing into robotic body materials and structures enables more autonomous, robust, and biocompatible monitoring and interventions in biomedical applications.

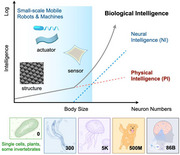

## 1 Introduction

**Figure 1:**
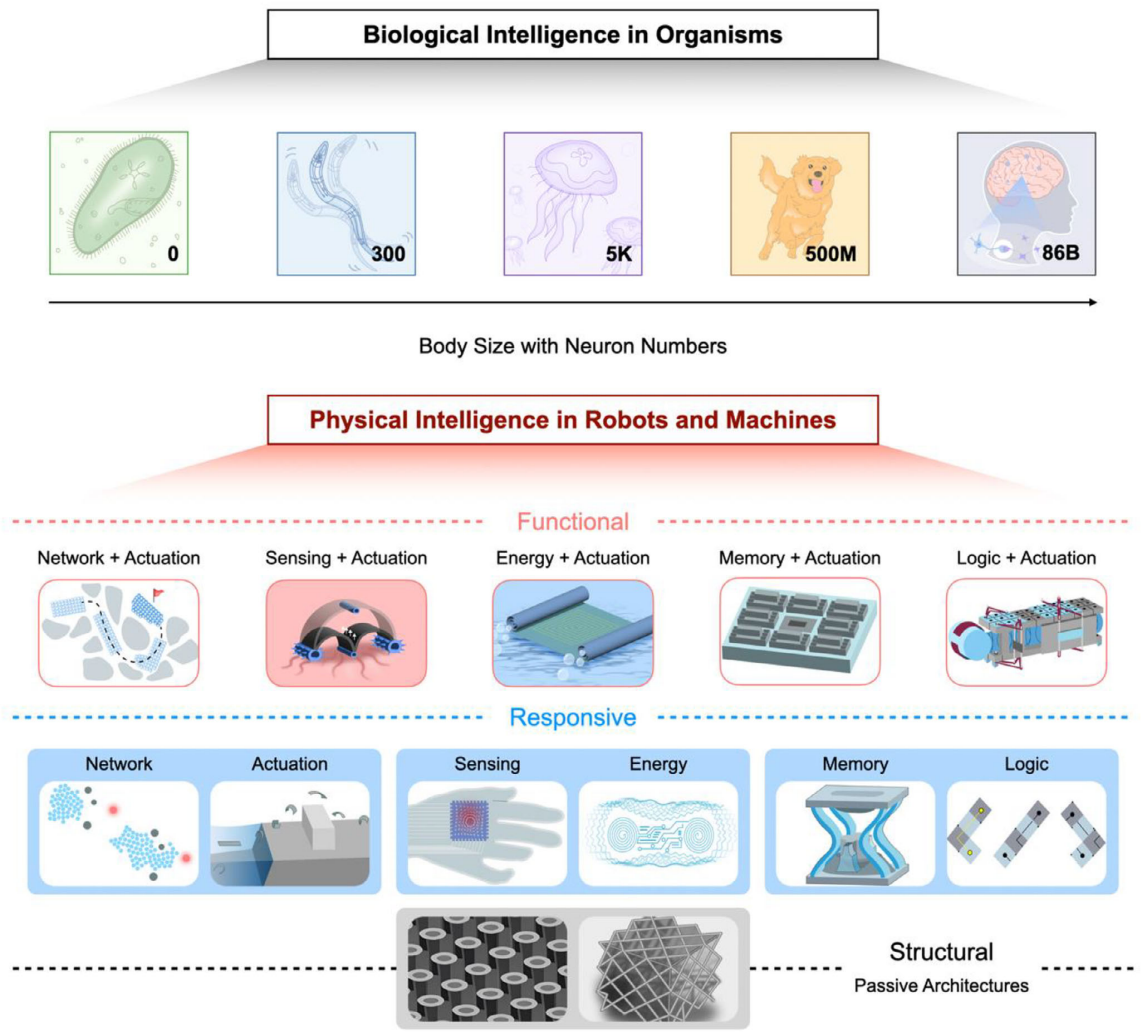
Intelligence in biological organisms and synthetic robotic systems. Inspired by biological intelligence at small scales, physical intelligence in artificial systems is the combination of “structural intelligence” and “material intelligence”, which can be subdivided into: i) Passive architectures exhibit inherent physical characteristics, such as adhesion, friction, strength, and stiffness. ii) Responsive materials, the foundation of soft actuators, flexible electronics, and active metamaterials, respond to external stimuli, such as force, pH, temperature, and chemicals. iii) Functional robots integrate the multiple characteristics mentioned above for specific real‐world applications. iv) Intelligent systems can interact with unstructured, dynamic, and complex environments through fully autonomous behaviors, while the creation of artificial machines comparable to natural organisms remains a knowledge gap.

Intelligence manifested in biological organisms, artificial machines, and mobile robots represents the ability to perceive (sense, interpret), control (decide, plan, predict, regulate), act (move, change, affect, coordinate), and learn (adapt, evolve, acquire experience, infer) continuously and automatically^[^ ^1^, ^2^, ^3^ ^]^ As shown in **Figure** **1**, taking single cells, plants, and some invertebrates as examples, physical intelligence (PI) dominates at small length scales. As the nervous system becomes increasingly complex, PI (the body), embodied intelligence (the body‐brain connection), and neural intelligence (the brain) together constitute advanced biological intelligence (BI) at large length scales.

The historical trajectory of man‐made machines empowered by PI can be traced back ≈3000 years, from ancient Greek “bronze automata”, to modern “strandbeest” artistic machines, and now extends toward next‐generation medical robots.^[^ ^4^, ^5^, ^6^ ^]^ To date, PI has expanded into a combination of “structural intelligence” and “material intelligence”, bringing higher impact to multidisciplinary researchers. At several tens of centimeters and meter length scales, the incremental integration of PI in their body serves to minimize the computational load, enhance energy efficiency and complementary functions, and finally simplify the robotic systems.^[^ ^7^, ^8^, ^9^ ^]^

At a smaller length scale (the longest feature ≤ 5 cm and the overall mass ≤ 5 g), PI works together with modern electronics to provide a “hybrid approach” for advanced intelligent robots.^[^ ^3^, ^10^ ^]^ Taking insect‐scale^[^ ^11^, ^12^, ^13^ ^]^ and capsule‐shaped robots^[^ ^14^, ^15^, ^16^ ^]^ as examples, the main challenge is how to achieve multimodal locomotion and perceive environmental changes under the severe constraints of onboard energy and communication distance. The optimization of mechanical mechanisms and the utilization of advanced materials can be the best strategies for centimeter‐scale robots, which is exactly what PI advocates.

At the other end of the spectrum, for micro/nanomachines, the priorities of dominant physical effects have undergone a fundamental shift.^[^ ^3^, ^17^ ^]^ Such differences can be quantitatively described by the “Scaling Law”, where viscous forces, surface forces, and Brownian motion replace inertial forces and gravity.^[^ ^18^, ^19^, ^20^ ^]^ Meanwhile, the mismatch of electronics imposes higher requirements on environmental responsive abilities, pushing PI to become the only core strategy to further advance the robotic intelligence.

In this review, we discuss the state‐of‐the‐art methods for leveraging PI capabilities at a scale of several centimeters and below. Next, we summarize diverse real‐world applications and current challenges for PI‐based robots and machines in extreme environments. Finally, we highlight promising technologies for further integrating PI with robotics, expecting breakthroughs from fundamental science to biomedical translation.

## 2 Small‐Scale Passive Architectures

**Figure 2:**
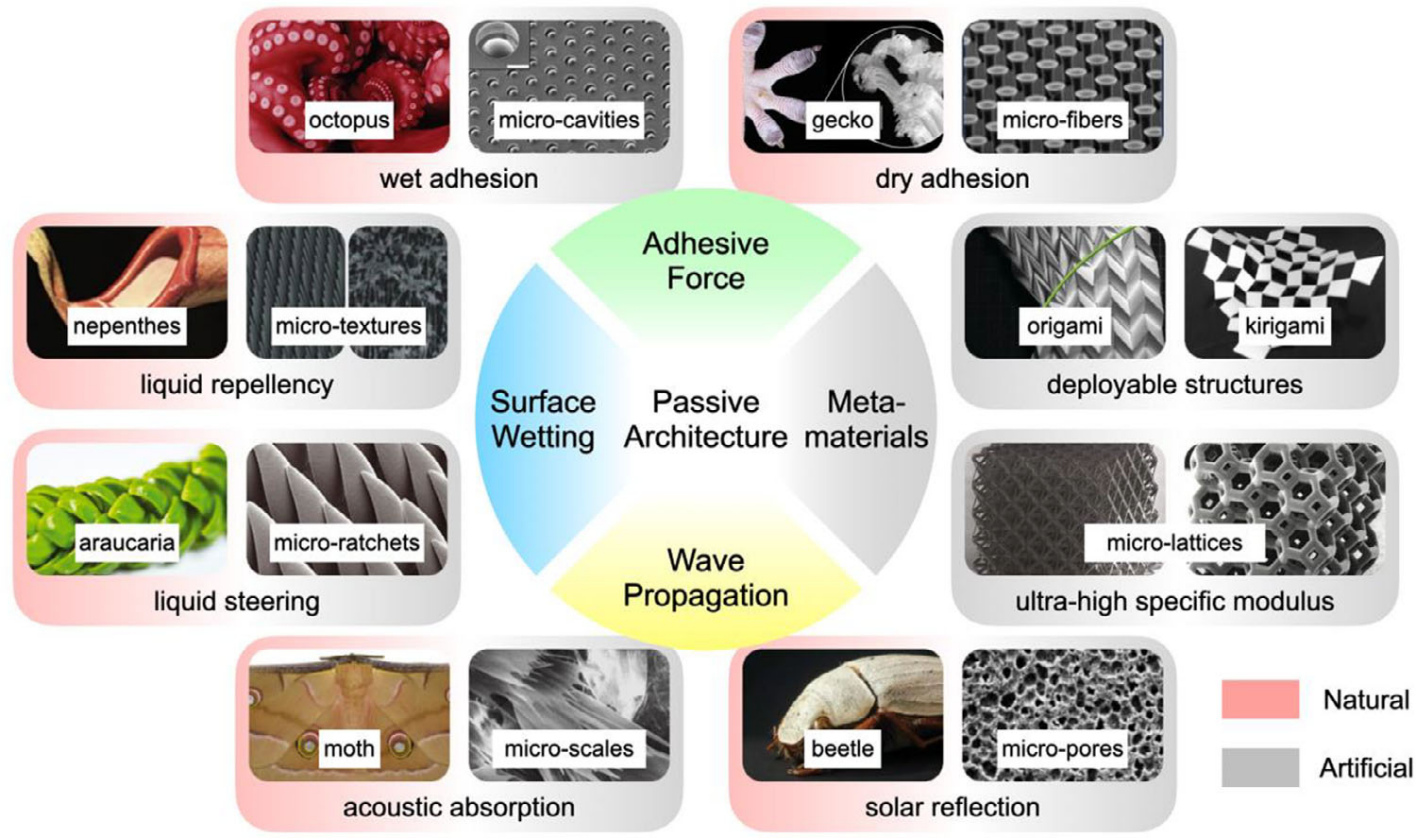
Physical intelligence enabled by small‐scale passive architectures.

A common strategy for creating PI at small scales involves taking inspiration from biological materials, structures, or mechanisms, as shown in **Figure** **2**. On one hand, geckos are renowned for their exceptional ability to adhere and climb to almost any surface, a trait generally attributed to the millions of fine hairs on their feet.^[^ ^21^, ^22^ ^]^ Drawing inspiration from these hairs, synthetic dry adhesives have been developed that incorporate mushroom‐shaped microfibers, significantly enhancing and controlling adhesion and friction.^[^ ^23^, ^24^ ^]^ On the other hand, soft suction cups inspired by octopi and remoras,^[^ ^25^, ^26^ ^]^ as well as hydrogels inspired by mussels and barnacles,^[^ ^27^, ^28^ ^]^ provide ingenious solutions for wet adhesion. These PI‐based adhesion strategies have flourished and made contributions to various fields such as semiconductor manufacturing, soft grippers, miniature robots, and tissue repair.^[^ ^5^, ^29^, ^30^, ^31^ ^]^

Inspired by nature with advanced single or multiple functions, artificial architectures are endowed with unconventional mechanical, fluidic, acoustic, and optical properties. Reproduced with permission^[^ ^21^ ^]^ Copyright 2000, Spring Nature;^[^ ^25^ ^]^ Copyright 2017, Spring Nature;^[^ ^32^ ^]^ Copyright 2016, Spring Nature;^[^ ^33^ ^]^ Copyright 2011, Spring Nature;^[^ ^34^ ^]^ Copyright 2021, The American Association for the Advancement of Science;^[^ ^35^ ^]^ Copyright 2018, National Academy of Sciences;^[^ ^36^ ^]^ Copyright 2023, The American Association for the Advancement of Science;^[^ ^37^, ^38^, ^39^ ^]^ Copyright 2014, The American Association for the Advancement of Science;^[^ ^40^ ^]^ Copyright 2019, Spring Nature. Reproduced under terms of the CC‐BY license.^[^ ^41^ ^]^

Plants also play a crucial role as embodiments of PI with their surface microstructures. Inspired by lotus leaves, nepenthes, and araucarias, superwettability phenomena emerge on various artificial surfaces, finding applications in hydrophobic coatings, anti‐fouling layers, and liquid steering.^[^ ^32^, ^33^, ^34^, ^42^, ^43^ ^]^ Besides, mechanisms in moth scales, beetle exoskeletons, and mimosa leaves have been transformed into acoustic, optical, and vibrational metasurfaces to control the wave propagation.^[^ ^35^, ^36^, ^41^, ^44^, ^45^ ^]^

In contrast, mechanical metamaterials refer to artificially designed structures that exhibit properties not found in conventional materials, such as negative Poisson's ratio, ultra‐light weight, and ultra‐high stiffness.^[^ ^37^, ^38^, ^39^, ^40^, ^46^ ^]^ With the advent of big‐data models and more comprehensive databases,^[^ ^47^, ^48^, ^49^ ^]^ computational design and simulation tools can aid in the performance evaluation and inverse design. Multistable, non‐reciprocal, and anisotropic mechanical metamaterials have garnered widespread interest in fields including shock absorption, fracture resistance, and mechanical computing.^[^ ^50^, ^51^, ^52^, ^53^, ^54^ ^]^

## 3 Small‐Scale Soft Actuators

Stimuli‐responsive materials play a pivotal role in small‐scale systems by enabling sensing, morphing, locomotion, logic, and memory. Distinguished from traditional, rigid, and bulky robots, small‐scale soft actuators can adapt to diminutive, dynamic, and dangerous environments, making them advantageous for encoding more advanced PI in their physical agents.^[^ ^55^, ^56^, ^57^ ^]^ Fluid‐driven soft actuators change their shape based on the pressure of cavities in polymers,^[^ ^58^, ^59^, ^60^ ^]^ but they are difficult to miniaturize down to millimeter scales, and cannot always get rid of air pumps, valves, or tubes.

**Figure 3:**
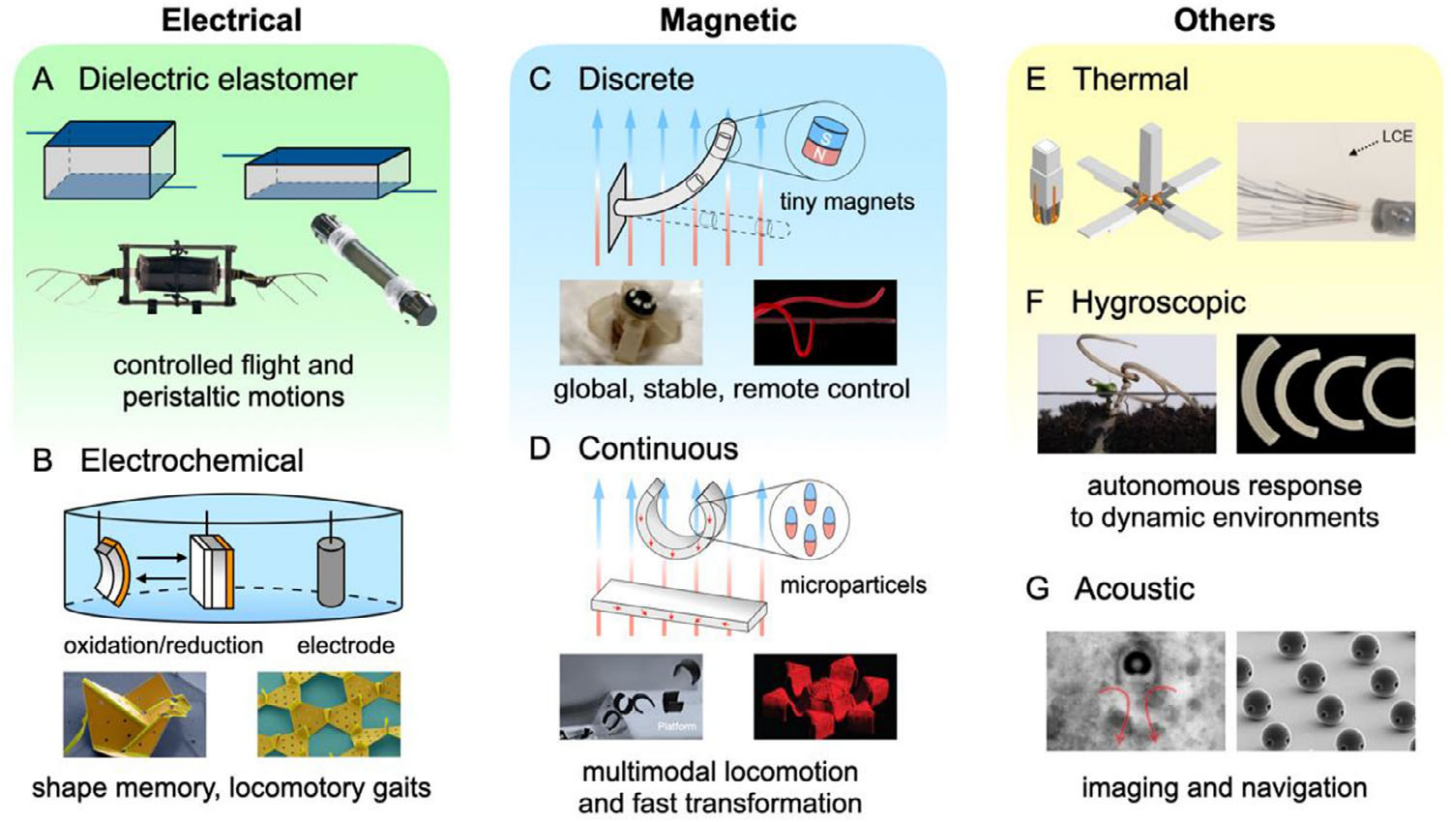
Physical intelligence exhibited through small‐scale soft actuators. A) Dielectric elastomer actuators undergo significant deformation under thousands of Volts. Reproduced with permission^[^ ^61^ ^]^ Copyright 2019, Spring Nature;^[^ ^65^ ^]^ Copyright 2022, The American Association for the Advancement of Science. B) Surface electrochemical actuators change the surface stress in the surrounding aqueous electrolyte. Reproduced with permission^[^ ^68^ ^]^ Copyright 2021, The American Association for the Advancement of Science;^[^ ^69^ ^]^ Copyright 2024, Spring Nature. C) Discrete magnetic actuators feature highly concentrated and localized magnetic moments in the soft shells. Reproduced with permission^[^ ^72^ ^]^ Copyright 2012, IEEE; Reproduced under terms of the CC‐BY license.^[^ ^73^ ^]^ D) Continuous magnetic actuators can have pre‐programmed distributed magnetic moments in the soft composites. Reproduced with permission^[^ ^74^, ^75^ ^]^ Copyright 2018, Spring Nature. E–G) Other types of small‐scale soft actuators also include thermal, optical, hygroscopic, and acoustic options,^[^ ^76^, ^77^, ^78^ ^]^ depending on specific requirements. Reproduced with permission^[^ ^79^ ^]^ Copyright 2021, The American Association for the Advancement of Science;^[^ ^80^ ^]^ Copyright 2023, Spring Nature;^[^ ^81^ ^]^ Copyright 2022, Spring Nature;^[^ ^78^ ^]^ Copyright 2024, The American Association for the Advancement of Science. Reproduced under terms of the CC‐BY licenses.^[^ ^76^, ^82^ ^]^

As shown in **Figure** **3**, we summarize several state‐of‐the‐art works of small‐scale soft actuators according to their principles and evaluate their advantages and challenges. Chen and colleagues drive robotic bees using dielectric elastomer actuators (DEAs), which have a power density of 600 watts per kilogram and can withstand collisions and damage.^[^ ^61^, ^62^, ^63^ ^]^ The environmental adaptability of DEAs is also evident in various climbing robots.^[^ ^64^, ^65^ ^]^ Hydraulically amplified self‐healing electrostatic (HASEL) actuators incorporate the advantages of DEAs and fluid‐driven soft actuators, enabling artificial muscles with impressive force outputs.^[^ ^66^ ^]^ Inevitably, both DEAs and HASELs require very high voltages (kV), which trigger not only safety issues, but also residual charge and miniaturization difficulties. To deal with such issues, low‐voltage actuation mechanisms have been proposed. Through electrochemical oxidation/reduction of a platinum surface, tethered bending micrometer‐scale actuators can function as morphing machines.^[^ ^67^, ^68^, ^69^ ^]^ Recently, for skin‐integrated haptic interfaces, electromagnetic millimeter‐scale actuators have become promising alternatives with better dexterity.^[^ ^70^, ^71^ ^]^

The emergence of untethered soft actuators has led to a new technological breakthrough in small‐scale robotics. In particular, natural tissues and organs are transparent to low‐frequency magnetic fields, and magnetic soft actuators can be remotely, accurately, and rapidly modulated without causing any adverse effects on biological systems.^[^ ^57^, ^83^ ^]^ Depending on the composite structure, we classify magnetic soft actuators into either discrete (a few tiny magnets)^[^ ^72^, ^73^, ^84^ ^]^ or continuous systems (embedded micro‐particles).^[^ ^74^, ^75^, ^85^ ^]^ Most impressively, recent works demonstrated unprecedented multimodal locomotion and fast transformation in three dimensions,^[^ ^74^, ^75^ ^]^ laying the foundation for this emerging field. To date, magnetic medical robotics research has grown exponentially in both engineering and medical journals.^[^ ^86^ ^]^ In the coming decades, researchers will aim to identify practical medical scenarios, conduct in vivo animal trials, and promote the clinical translation of such robots.

For different tasks, there are also many other options: Environmental temperature and humidity changes can serve as energy sources for liquid crystal elastomers (LCEs), shape‐memory polymers (SMPs), hydrogels, and cellulose,^[^ ^79^, ^80^, ^81^, ^82^, ^87^ ^]^ bringing small‐scale robots in the wild one step closer to full autonomy. Benefiting from the rapid development of ultrasound technology—particularly the emergence of wearable ultrasound patches,^[^ ^88^, ^89^ ^]^ acoustic microswimmers^[^ ^76^, ^77^, ^78^ ^]^ are expected to integrate imaging and navigation to achieve radiation‐free, real‐time, and closed‐loop surgical interventions.

## 4 Small‐Scale Computational Networks

Mechanical computing is another typical demonstration of PI, emphasizing mechanical transmission, artificial architectures, and the non‐Von Neumann framework as its core.^[^ ^53^, ^90^ ^]^ It implements Boolean operations^[^ ^91^, ^92^, ^93^ ^]^ and neural networks^[^ ^94^, ^95^, ^96^ ^]^ on specific external inputs (such as contact force, magnetic field, and thermal signals) to obtain detectable outputs (such as displacement, stiffness, and temperature), all while bypassing any electronics. The main limitation at present is that complex mechanisms, like arithmetic machines, cannot yet be miniaturized.

**Figure 4:**
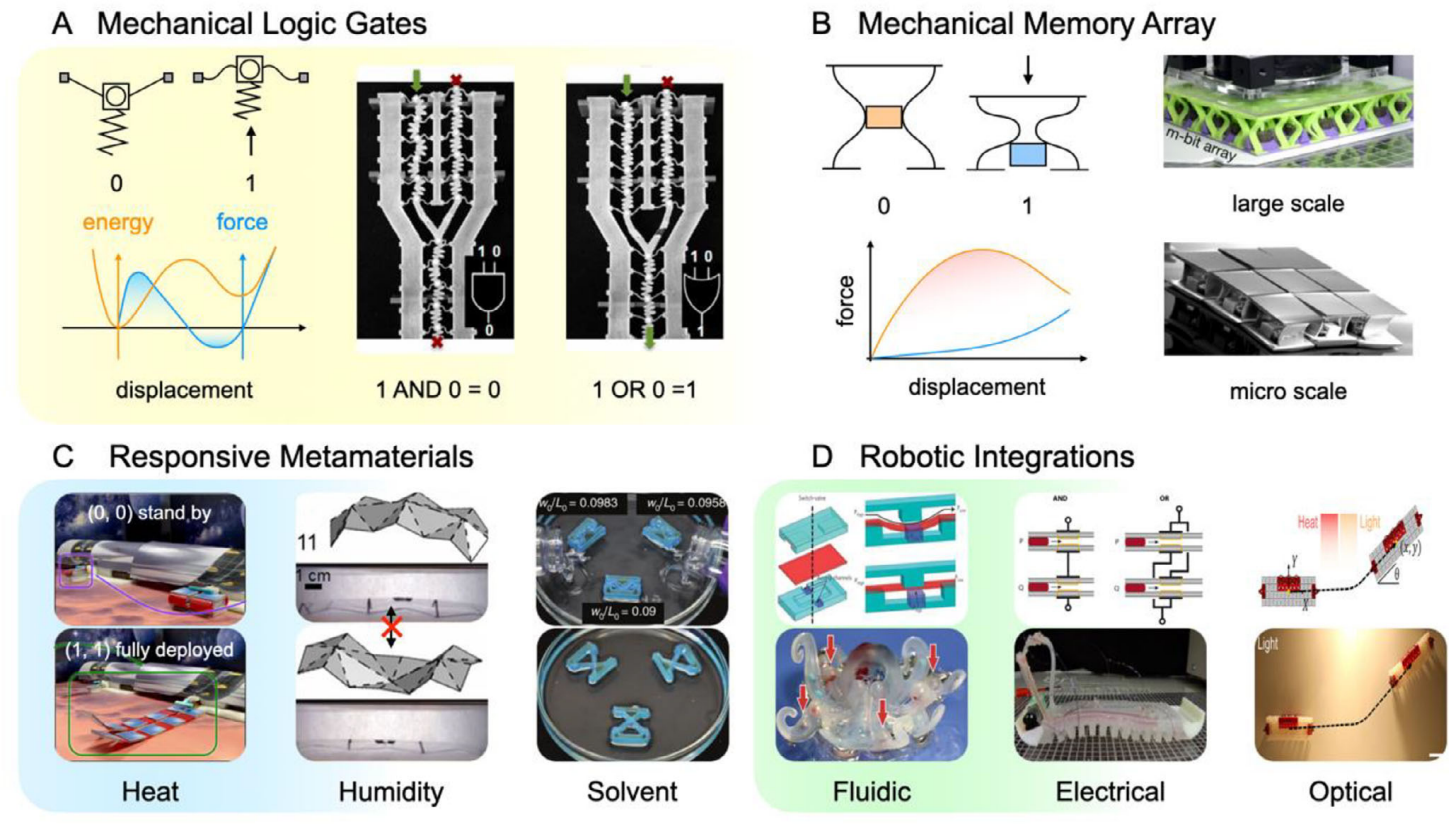
Physical intelligence encoded in mechanical computational networks. A) Serially connected mechanical bytes form binary logic gates. Reproduced with permission^[^ ^91^ ^]^ Copyright 2016, National Academy of Sciences. B) Parallelly connected mechanical bytes form programmable memory arrays. Reproduced with permission^[^ ^92^ ^]^ Copyright 2019, Spring Nature. Reproduced under terms of the CC‐BY license.^[^ ^100^ ^]^ C) Embedded soft actuators facilitate responsive metamaterials to adapt to environmental changes. Reproduced with permission^[^ ^101^ ^]^ Copyright 2024, John Wiley and Sons. Reproduced under terms of the CC‐BY license.^[^ ^102^, ^103^ ^]^ D) Embedded logic gates facilitate robotic integrations to make decisions autonomously. Reproduced with permission^[^ ^104^ ^]^ Copyright 2010, Spring Nature;^[^ ^105^ ^]^ Copyright 2016, Spring Nature;^[^ ^106^ ^]^ Copyright 2019, The American Association for the Advancement of Science. Reproduced under terms of the CC‐BY license.^[^ ^107^ ^]^

As shown in **Figure** **4**, several representative mechanisms of mechanical computing and related applications are listed. Mechanical bytes are the basis of unconventional computing networks, usually connected in series to construct logic circuits and in parallel to store binary data. Buckled beams are customized to store or release nonlinear elastic energy, yielding logic gates and memory arrays;^[^ ^91^, ^92^ ^]^ Rotating tiles, which are determined by the connection or absence of conductive traces, are more suitable for running arithmetic operations.^[^ ^97^, ^98^ ^]^ Recently, some impressive attempts have demonstrated micro‐scale mechanical bytes,^[^ ^99^, ^100^ ^]^ whose dynamic performance is expected to be systematically characterized.

The main intentions behind the revival of mechanical computing are as follows:

- To provide an alternative to the failure of electronics in extreme environments, such as ultra‐high magnetic fields, strong nuclear radiation, corrosive gases/liquids, and drastic temperature differences.^[^ ^53^, ^101^, ^102^, ^103^ ^]^ Although specialized encapsulation can protect electronics to a certain extent, it also blocks the electronics from perceiving and interacting with dynamic environments.
- To alleviate the burden of feedback, control, and energy supply on autonomous systems, especially in specific missions. An increasing number of electronics‐free robots have proven their efficiency with unconventional technologies, including versatile grasping, trajectory changing, and communication protocols.^[^ ^104^, ^105^, ^106^, ^107^, ^108^, ^109^ ^]^
- To clarify unprecedented fundamental science via 3D metamaterials. Notably, chiral, non‐commutative, and non‐reciprocal mechanical metamaterials^[^ ^110^, ^111^, ^112^ ^]^ are being developed for mechanical logic gates and memory arrays.

## 5 Small‐Scale Collective Behaviors

**Figure 5:**
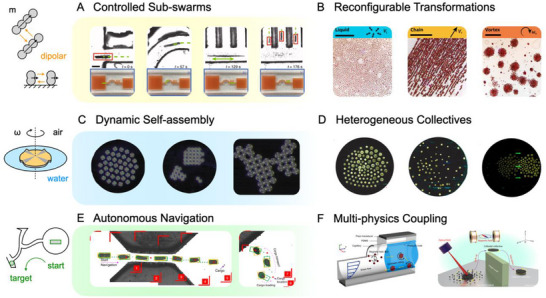
Physical intelligence emerged from small‐scale collective behaviors. A) Paramagnetic nanoparticles perform elongation and splitting. Reproduced under terms of the CC‐BY license.^[^ ^116^ ^]^ B) Ferromagnetic colloidal particles transform between several configurations. Reproduced with permission^[^ ^117^ ^]^ Copyright 2019, The American Association for the Advancement of Science. C) Homogeneous micro‐rafts demonstrate programmable self‐assembly. Reproduced under terms of the CC‐BY license.^[^ ^118^ ^]^ D) Heterogeneous micro‐rafts are also validated under external oscillating magnetic fields. Reproduced under terms of the CC‐BY license.^[^ ^119^ ^]^ E) Deep learning guides a micro‐swarm to learn optimal distributions in unstructured morphologies. Reproduced with permission^[^ ^120^ ^]^ Copyright 2022, Spring Nature. F) The combinations of magnetic, acoustic, and optical fields enable more complex locomotion. Reproduced with permission^[^ ^121^ ^]^ Copyright 2021, Spring Nature. Reproduced under terms of the CC‐BY license.^[^ ^122^ ^]^

Due to the limited capabilities of individual micro/nanorobots, the collective behaviors of swarms, which utilize coupled physical interactions among the robots, are an effective method for creating PI.^[^ ^113^, ^114^, ^115^ ^]^ Among them, as shown in **Figure** **5**, magnetic swarms allow diverse collective behaviors at the cellular scale using coupled hydrodynamic, magnetic, capillary, or electrostatic long‐ or short‐range and attractive or repulsive physical interactions.

Programmable, reconfigurable, and directional morphing and locomotion are the most competitive features of magnetic swarms. A ribbon‐like paramagnetic nanoparticle swarm can form multiple sub‐swarms by increasing the input direction angle for the oscillating fields;^[^ ^116^ ^]^ another peanut‐shaped ferromagnetic microrobot swarm can provide versatile, fast, reversible transformations.^[^ ^117^ ^]^ When the diameter of the individual particle increases to 50 µm level, Wang and colleagues demonstrated dynamic self‐assembly of homogeneous micro‐rafts at the air‐water interface;^[^ ^118^ ^]^ in a similar platform, diverse reconfigurable collective modes of heterogeneous micro‐rafts with asymmetric pairwise interactions were further studied.^[^ ^119^ ^]^ Recent advances focus on the contribution of deep‐learning algorithms^[^ ^120^, ^123^ ^]^ and multi‐physics coupling^[^ ^121^, ^122^ ^]^ to design and control collective navigation behaviors. Such small‐scale collective swarms are desired robotic behaviors in environmental and medical applications, such as water purification, drug delivery, and real‐time tracking.^[^ ^124^, ^125^, ^126^ ^]^

## 6 Current Challenges and Future Opportunities

**Figure 6:**
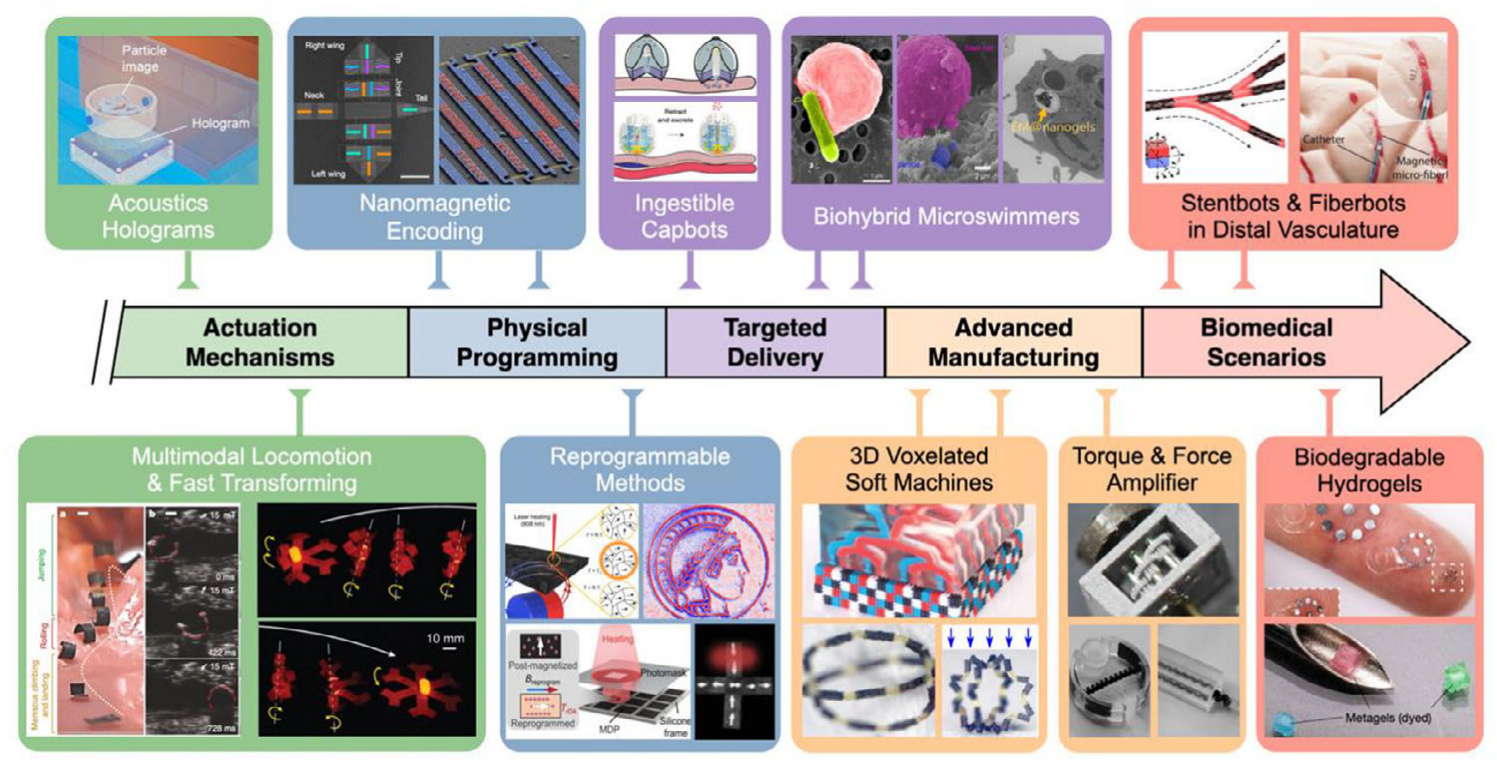
Evolutionary trajectory of PI in small‐scale robots and machines.

In the past decade, small‐scale robots and machines have traversed several distinct historical milestones on the voyage to advance their PI, evolving from passive structures to intelligent systems. As shown in **Figure** **6**, the pursuit of effective untethered actuation at small scales started with acoustic and magnetic fields,^[^ ^74^, ^75^, ^127^ ^]^ especially toward promising medical applications. Despite programmable actuators accomplishing preset missions quite well, machines that cannot be altered once fabricated met many shortcomings in complex, changing, and unstructured environments. Reprogrammable magnetization methods were proposed for magnetic actuators with reconfigurability and adaptability.^[^ ^128^, ^129^, ^130^, ^131^ ^]^

The subsequent stage was targeted drug delivery and autonomous navigation of biomedical tasks. A typical example, based on PI, was an ingestible self‐orienting capsule, which can attach to the gastric wall from any starting position and facilitate the submucosal injection of macromolecules.^[^ ^132^, ^133^, ^134^ ^]^ For commonly inaccessible and even invisible regions, biohybrid microrobots demonstrated their abilities in specific tasks, such as precise navigation to the bile duct and passing across the blood‐brain barrier.^[^ ^135^, ^136^, ^137^ ^]^ At the same time, the remarkable progress in micro/nano‐manufacturing technologies promoted the diversity of robots and machines. Heterogeneous voxels endowed small‐scale soft robots with higher‐dimensional and complex shape‐morphing capabilities.^[^ ^138^, ^139^ ^]^ Moreover, miniature gearboxes and artificial muscles were shown to significantly amplify the output forces and power density for specific applications.^[^ ^140^, ^141^ ^]^

Over the past decade, the exploration of PI on small scales began with actuation mechanisms. Then, it was followed by a period of rapid iteration in critical technologies, including (re‐)programmability, precise navigation, and multi‐material printing. At present, small‐scale actuators and sensors with advanced PI are poised to address real‐world challenges, especially applications of robots in the wild and clinic.^[^ ^142^, ^143^, ^144^, ^145^ ^]^ Reproduced with permission^[^ ^127^ ^]^ Copyright 2016, Spring Nature;^[^ ^74^, ^75^ ^]^ Copyright 2018, Spring Nature;^[^ ^135^ ^]^ Copyright 2018, The American Association for the Advancement of Science;^[^ ^128^, ^138^ ^]^ Copyright 2019, Spring Nature;^[^ ^132^ ^]^ Copyright 2019, The American Association for the Advancement of Science;^[^ ^133^ ^]^ Copyright 2021, Spring Nature;^[^ ^136^, ^137^, ^139^ ^]^ Copyright 2021, The American Association for the Advancement of Science;^[^ ^131^ ^]^ Copyright 2021, John Wiley and Sons;^[^ ^140^ ^]^ Copyright 2022, The American Association for the Advancement of Science;^[^ ^144^ ^]^ Copyright 2024, Spring Nature;^[^ ^129^, ^143^, ^145^ ^]^ Copyright 2024, The American Association for the Advancement of Science. Reproduced under terms of the CC‐BY licenses.^[^ ^130^, ^141^, ^142^ ^]^

More recently, PI‐based small‐scale robots are expanding the boundaries of their real‐world applications, particularly in extreme environments. For insect‐scale robots in the wild, the integrations of artificial muscles (dielectric, piezoelectric, electrothermal, and electromagnetic soft actuators) and their output amplification machines (composite joints, resonance, weaving, and bistable flexible frames)^[^ ^61^, ^146^, ^147^, ^148^ ^]^ have replaced traditional motors and demonstrated performance that can surpass that of biological organisms. Meanwhile, flexible electronics provide key technologies such as conformal interfaces, decoupled sensing, and wireless communication,^[^ ^149^, ^150^, ^151^, ^152^ ^]^ outlining a blueprint for a “hybrid approach” for fully autonomous systems. It is also worth emphasizing that more and more insect‐scale robots have leveraged PI to transform environmental constraints into their empowering sources, such as self‐deployment, self‐oscillation, and self‐morphing mechanisms.^[^ ^153^, ^154^, ^155^ ^]^

For medical robots tailored to clinical needs, we emphasize that the in‐vivo environment for large‐animal and human trials is extreme. First, robots must be small enough to enter the human body minimally invasively, while also large enough to integrate multifunctional modules. For ingestible robots in the gastrointestinal tract, the maximum diameter is 10 mm, and the longest side length is 25 mm (000#); while for implantable robots in the cerebrovascular system, their body size is limited to below the millimeter scale. Then, multiple barriers, such as a variety of digestive fluids (gastric juice pH < 2), intestinal mucus (thickness 10–100 µm and viscosity 10–1000 cP), the blood‐brain barrier (permeability coefficient of macromolecules <1 × 10^−^⁸ cm s^−1^), and dense cell clusters (gap width < 1 nm), seriously compromise the effectiveness of monitoring and treatment.^[^ ^156^, ^157^, ^158^, ^159^ ^]^ Next, in addition to precise targeted delivery, the actuation challenges of medical robots also include resisting the effects of respiration (10–20 breaths per minute), peristaltic waves (0.5–2 cm s^−1^ and 0–15 mmHg intraluminal pressure),^[^ ^26^, ^160^, ^161^ ^]^ blood flow (30–80 cm s^−1^ and 60–100 mmHg in the cerebral artery),^[^ ^121^, ^142^, ^143^ ^]^ and other factors. Finally, due to the stringent constraints on size, power consumption, and communication distance for batteries, antennas, and chips, the sensing issues in deep tissue remain unsolved. Recent breakthroughs come from biodegradable stimuli‐responsive hydrogels: based on the metamaterials and shape‐adaptive structures, ultrasonic imaging can provide key physiological and pathological indicators such as pressure, strain, temperature, and pH.^[^ ^144^, ^145^, ^162^ ^]^ Smarter, smaller, softer, safer robots are reshaping biomedical engineering through untethered in‐vivo manipulation and long‐term wireless monitoring.

**Figure 7:**
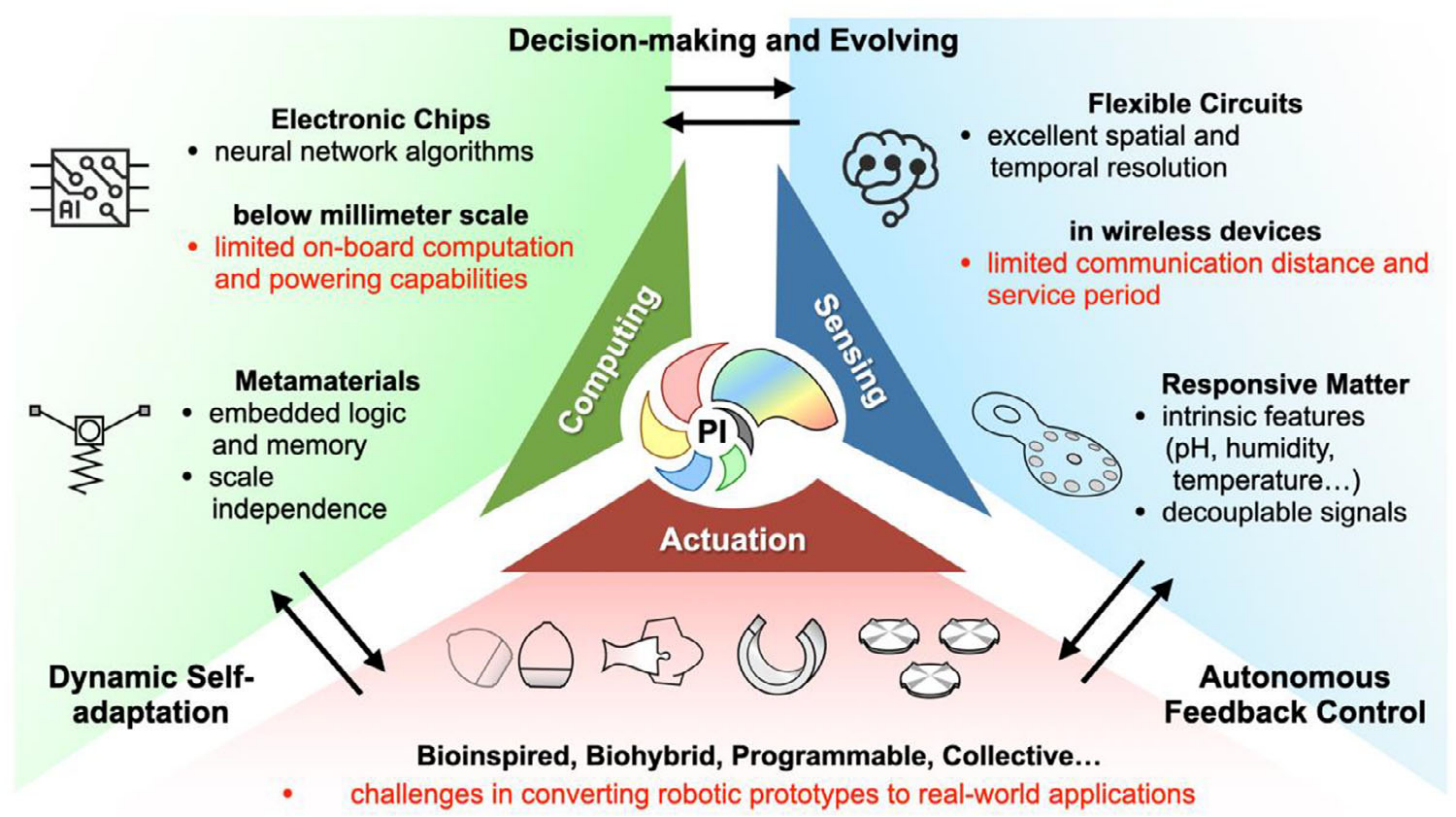
Strategies to promote PI in small‐scale robots and machines.

Physical intelligence emphasizes a new paradigm of “structural intelligence” + “material intelligence” to seamlessly integrate computing, sensing, and actuation, thereby optimizing the interactions of small‐scale robots and machines in complex real‐world environments.

In the following decades, PI is expected to achieve more complex and sophisticated functional integration, further propelling small‐scale robots toward intelligence and autonomy. As shown in **Figure** 7, we put forward the promising strategies to promote PI:

(i) **Decision‐making and evolving**: Biological systems, particularly unicellular organisms and plants, can make decisions conducive to their survival, all without the presence of a nervous system.^[^ ^3^ ^]^ Technically, even for insect‐scale and capsule robots, they only have the volume equivalent to two button batteries, which cannot support the power consumption of complex processors—let alone sub‐millimeter‐scale devices. Another extra issue can be that batteries account for the vast majority of the weight and volume, and pose potential safety hazards of leakage and overheating.^[^ ^163^, ^164^, ^165^ ^]^ Battery‐free wireless energy transfer and communication technologies only have a coverage range of a few centimeters and attenuate to undetectable levels within a few millimeters in in vivo environments.^[^ ^166^, ^167^, ^168^ ^]^ Thus, there remains a trade‐off between on‐board computing and effective distance.

Compared to traditional electronic chips, mechanical neural networks can encode logic, memory, and learning functions into robotic bodies. Exciting examples at the macroscale have demonstrated the feasibility of mechanical structures in backpropagation, learning, and in‐memory computing.^[^ ^94^, ^95^, ^96^ ^]^ Moreover, due to the scale independence of metamaterials, smaller structures should exhibit similar properties. The ideal workflow will be as follows: micro‐sensors receive external stimuli, mechanical neural networks run algorithms to make decisions, and through comparison with sensing data from different time points, they can achieve self‐evolution in extreme environments.

(ii) **Dynamic self‐adaptation**: Traditional small‐scale robots and machines rely on preprogrammed properties and lack efficient interactions with complex, unstructured, and dynamic environments. While operating in parallel with the decision‐making process, PI also emphasizes dynamic self‐adaptation to regulate robotic morphology, trajectory, and locomotion. On one hand, the construction of robots with various metamaterials has garnered widespread interest, such as kirigami, origami, and lattices,^[^ ^69^, ^110^, ^169^ ^]^ enabling them to be more agile, robust, lighter, and faster.

On the other hand, how to quantitatively characterize the dynamic properties of active metamaterials is an emerging research field. Over the past decade, the static mechanical properties of metamaterials have been well characterized,^[^ ^38^, ^39^, ^50^ ^]^ while dynamic fracture, optical diffraction, and acoustic impedance^[^ ^170^, ^171^, ^172^ ^]^ during actuation will become increasingly important in the future. Physical analog computing circuits, high‐density memory arrays, and adaptive robotic controllers will all benefit from the development of dynamic metamaterials.

(iii) **Autonomous feedback control**: The autonomy of medical robots can be categorized into the following levels:

Robotic assistance: Human operator maintains continuous control;

Task autonomy: Operators have discrete control over a specific task;

Conditional autonomy: Generating different strategies, with selection made by a human operator;

High autonomy: Making decisions under the supervision of a qualified physician;

Full autonomy.^[^ ^173^ ^]^

At the current stage, small‐scale robots remain below Level 3. Employing stimuli‐responsive materials catalyzes the seamless integration of actuation and sensing functions. Untethered actuators and wireless sensors will work together to assist in the precise navigation, position correction, and dynamic scanning of the medical implants. Notably, threshold setting and orthogonal decoupling are particularly critical. Taking liquid crystal elastomers (LCEs) as an example,^[^ ^10^, ^87^, ^174^ ^]^ when used as drug carriers in vivo, they should neither be triggered below normal temperature (≈38 °C) nor be actuated beyond the tolerance limit (about 45 °C). In such scenarios, the mechanisms for targeted delivery or prolonged retention should rely on magnetic or pH‐responsive materials to avoid incorrect drug release. Autonomous small‐scale robots with in situ diagnosis‐therapy closed‐loop feedback will revolutionize the minimally invasive and non‐invasive interventions.

## 7 Perspective Roadmap for PI in Small‐Scale Medical Robots

**Figure 8:**
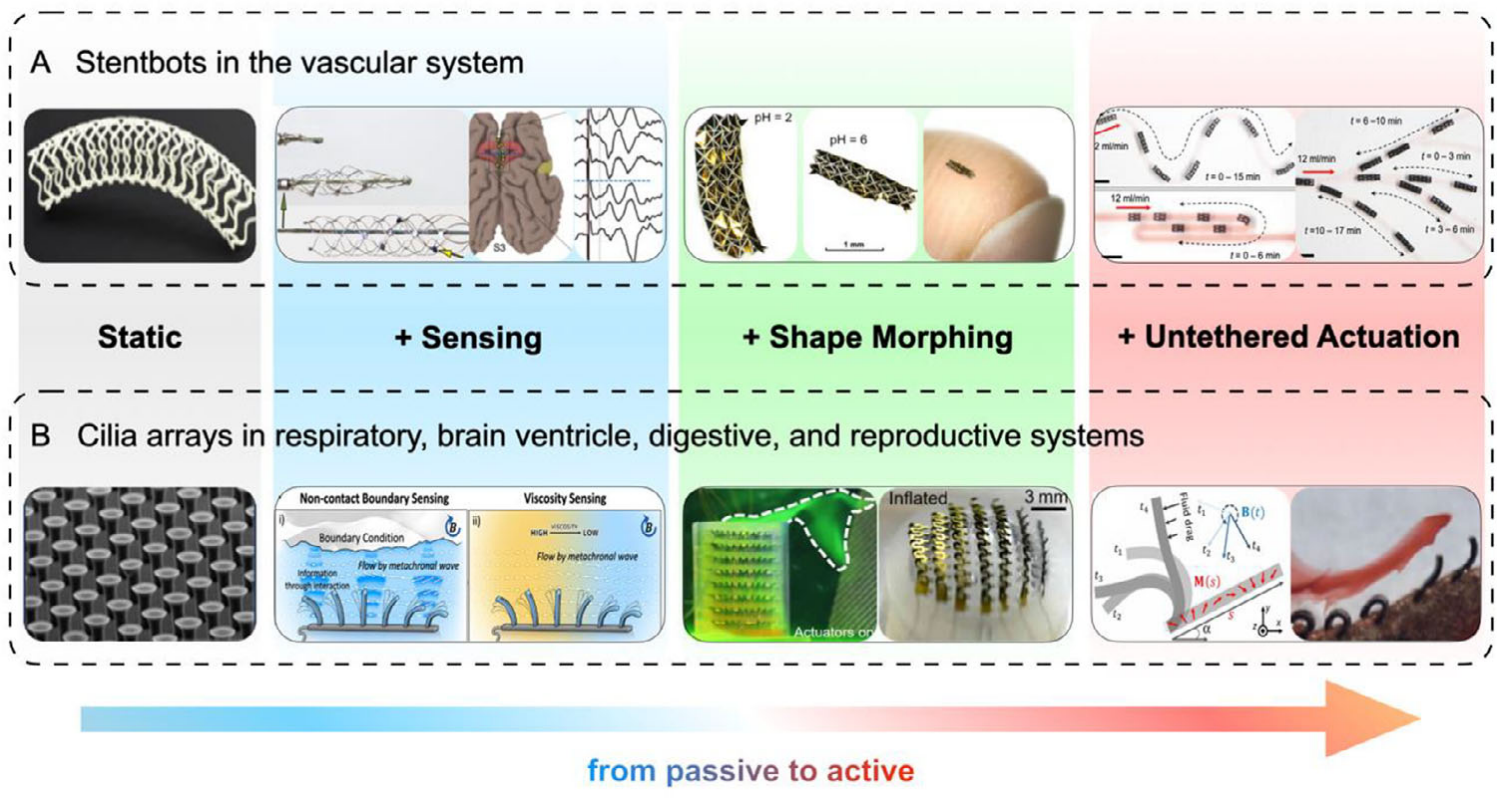
Roadmap for leveraging physical intelligence in small‐scale medical robots. A) Small‐scale stents in specific medical scenarios, including signal recording, deployable mechanisms, and remote navigation. Reproduced with permission^[^ ^175^, ^176^ ^]^ Copyright 2016, Spring Nature. Reproduced under terms of the CC‐BY licenses.^[^ ^142^, ^177^ ^]^ B) Small‐scale cilia arrays with various fluid manipulation and sensing functions on flat or curved surfaces. Reproduced under terms of the CC‐BY licenses.^[^ ^178^, ^179^, ^180^ ^]^

To illustrate how to leverage PI in small‐scale robots and machines, we present roadmaps for two typical biomedical scenarios, as shown in **Figure** **8**. For extreme in vivo environments, robot prototypes should be highly compatible with approved devices, imaging systems, and surgical procedures to facilitate animal model trials and subsequent clinical translation. One of the best ways to foster creativity is to work within a set of strict constraints, and the best such constraints are those posed by the human body and clinicians.

Stents, designed to support narrowed or blocked luminal structures in the body, are one of the most commonly used interventional methods for cardiovascular and cerebrovascular diseases.^[^ ^175^, ^181^ ^]^ Recent advances have reported a passive stent‐electrode array (stentrode) that can record brain activity from within a vein.^[^ ^176^, ^182^ ^]^ Modified commercial Nitinol stents were implanted into the 2.4 mm‐diameter superior sagittal sinus with the assistance of X‐ray and catheters. Using micro‐origami tessellation technology,^[^ ^177^, ^183^ ^]^ foldable and deployable stents were also validated. However, none of the aforementioned stents can achieve precise navigation, especially in the distal vascular system. Wang and colleagues proposed a wireless stent‐shaped magnetic soft robot to be deployed, actively navigated, and retrieved in the 1 mm‐diameter M4 segment of the middle cerebral artery.^[^ ^142^, ^184^ ^]^

**Figure 9:**
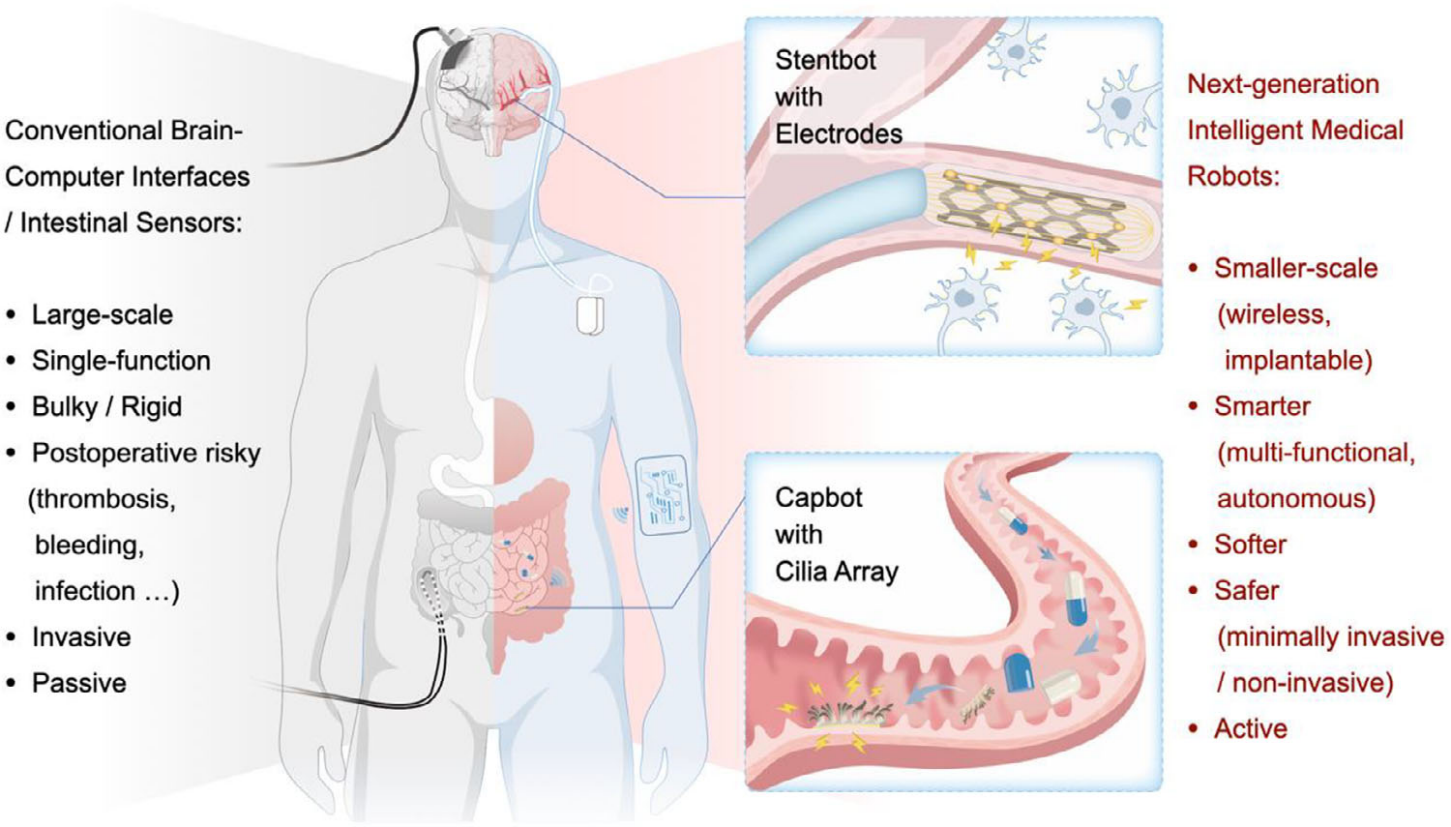
Perspectives for next‐generation intelligent small‐scale medical robots.

As shown in **Figure** **9**, we anticipate that the next‐generation intelligent medical robots will closely integrate “structural intelligence” and “material intelligence” to achieve closed‐loop monitoring and treatment in extreme in‐vivo environments. Small‐scale PI‐based robots hold significant potential to lead a revolution in the field of brain‐computer interfaces (BCIs). Magnetic stents,^[^ ^142^, ^184^ ^]^ helixes,^[^ ^143^, ^185^ ^]^ and guidewires^[^ ^85^, ^186^ ^]^ can precisely deliver flexible electrodes^[^ ^187^, ^188^, ^189^ ^]^ into distal blood vessels at the millimeter or even submillimeter scale, and ultimately anchor them effectively at the correct bifurcation sites to continuously record and stimulate peripheral nerves.

Compared with conventional invasive interventions, smaller‐scale, smarter, softer, and safer robots will make people's lives better. Two proposals demonstrate the PI‐based highly integrated robots in the distal vasculature and deep intestinal areas.

Another example of PI‐based machines is the bioinspired microfibers, which have been extended to cilia arrays, serving as an effective platform to inherit more functions and improve intelligence. With fluid–cilia interactions, artificial cilia perceived the surrounding fluidic environment, such as the boundaries and viscosity.^[^ ^178^, ^190^ ^]^ Then, several photo‐responsive^[^ ^191^, ^192^ ^]^ and electrically driven cilia arrays provided higher degrees of operational freedom.^[^ ^179^, ^193^ ^]^ Through programmable magnetization, Dong and colleagues achieved both non‐reciprocal motion and metachronal coordination with artificial cilia arrays.^[^ ^180^, ^194^ ^]^

The key challenge lies in converting robotic prototypes to real‐world applications. Ingestible capsules^[^ ^14^, ^15^, ^26^ ^]^ can serve as a considerable carrier to implant cilia arrays into the deep regions of the GI tract. Artificial cilia arrays will contribute to the dynamic regulation of the mucus layer, the directional transport of intestinal contents, and mechanical and chemical sensing for homeostasis, through remote control and wireless communication.

## 8 Conclusion

Physical Intelligence has been progressing fast recently in the realm of small‐scale robots and machines. From passive architectures to mobile robots, PI has demonstrated its uniqueness, advancement, and practicality across diverse dimensions. At the centimeter scale and above, PI shields redundant information, simplifies control circuits, and improves robot performance by coordinating with electronics. At the millimeter scale and below, PI dominates due to the miniaturization limitations of onboard computational systems. On one hand, PI robots implement the specific “perception‐processing‐actuation‐feedback” loops through programmable mechanisms, metamaterials, and stimuli‐responsive materials. On the other hand, many roboticists have initially got inspiration from nature (morphology, locomotion, and functions) when creating PI on small scales. In subsequent iterative optimizations, PI has deepened the understanding of natural evolution and advanced the fundamental science. Most promisingly, for the extreme in‐vivo inner‐body environments, PI will bring better safety, longer‐term stability, and higher cost‐effectiveness to implantable and ingestible medical robotic systems.

## Conflict of Interest

The authors declare no conflict of interest.
